# Towards time-resolved multiscale and multimodal imaging

**DOI:** 10.1038/s44303-025-00131-w

**Published:** 2025-12-24

**Authors:** Jishizhan Chen

**Affiliations:** Multiscale X-ray Imaging (MXI) lab, Department of Mechanical Engineering, University College London, London, UK

**Keywords:** Computational biology and bioinformatics, Diseases, Health care, Mathematics and computing, Medical research

## Abstract

Current biomedical imaging focuses on spatial detail but overlooks time, limiting our understanding of disease progression. There is an unmet need for temporal atlases that align multiscale and multimodal data across defined timepoints, enabling dynamic mapping of pathophysiology. This framework will pave the way for more personalised, time-aware diagnostics and interventions.

## Biomedical Imaging Remains Trapped in Spatial Resolution

Biomedical imaging has long prioritised spatial resolution, enabling us to visualise the human body across multiple scales and modalities. These capabilities have revolutionised the way we diagnose and study disease. Despite these advances, most imaging remains in space rather than time. It captures static snapshots, offering little insight into how biological processes take place.

This reflects a fundamental limitation. Disease is dynamic by nature. Tissues remodel gradually, tumours grow over weeks or months, and neurodegeneration progresses over years. Without temporal information, we miss the sequence of changes that shape disease. By relying on isolated timepoints, we may identify damage but fail to understand how it developed. Imaging studies may include different disease stages, but timepoints are usually disconnected and opportunistic. This lack of systematic temporal design limits our ability to track progression or intervene at pivotal moments.

**Fig. 1:**
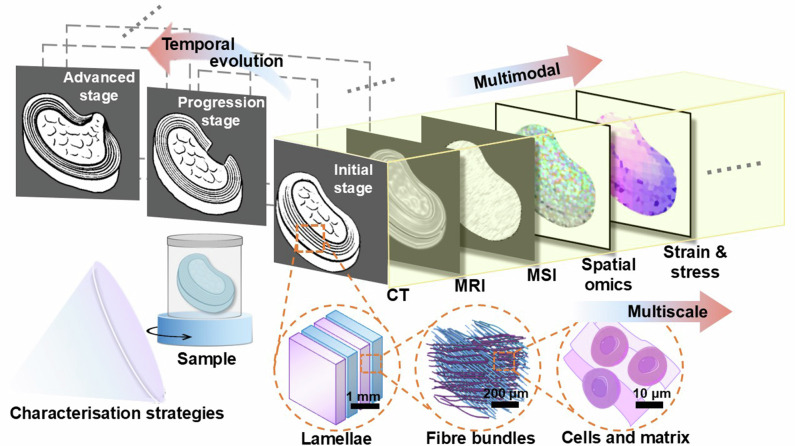
A framework for integrating multiscale and multimodal imaging with temporal evolution. Using intervertebral disc herniation as an example, the schematic showing how temporally anchored stages of disc degeneration can be characterised through integrated imaging. Time anchors include the initial stage (microstructural weakening), progression stage (structural instability), and advanced stage (extrusion and structural collapse). Multimodal approaches, including CT, MRI, mass spectrometry imaging (MSI), spatial omics, and strain/stress mapping, provide complementary anatomical, biochemical, molecular, and mechanical insights. In parallel, multiscale imaging resolves features across spatial hierarchies from millimetre-scale lamellar organisation to micrometre-scale collagen fibre bundles and cellular matrix components. Together, these strategies support the construction of time-resolved atlases that link structure, function, and molecular state across disease evolution.

Today’s multiscale imaging methods reveal structure from the organ to the cellular level, while multimodal strategies combine molecular, structural, and functional signals. However, these techniques rarely capture how features evolve over time (Fig. 1). Time is often treated as an external factor rather than a core dimension of analysis. Several dynamic imaging modalities already provide valuable temporal information, such as Doppler ultrasound, contrast-enhanced CT/MRI, and calcium or fluorescence imaging. These approaches capture fast physiological dynamics within a single modality. However, these approaches operate at one spatial scale and over short timeframes, and therefore do not address the longitudinal progression of disease. The temporal framework proposed here focuses on this longer-term, multiscale evolution, which current dynamic imaging cannot integrate. A few spatio-temporal models have been raised such as the Alzheimer’s Disease Course Map^1^, developmental spatio-temporal white matter atlases^2^, and omics-based trajectory inference frameworks^3^. Although they highlighted the importance of temporal dynamics, these approaches remain largely disease-specific, modality-restricted, or based on inferred pseudotime rather than real biological timepoints.

To move forward, imaging must begin to consider time as fundamental as space. Temporal disease atlases should be the emphasis at the next stage. Datasets connect not only multiple imaging resolutions and modalities, but also across biologically meaningful timepoints. Such atlases could reveal how structural, functional, and molecular features co-evolve, supporting earlier diagnosis, more accurate prognosis, and dynamic disease modelling. Integrating time as the fourth dimension is essential to bridge the gap between foundational mechanisms and clinical decision-making.

This perspective is therefore based on the hypothesis that the primary limitation of current multiscale and multimodal imaging is not the lack of spatial or molecular detail, but the absence of a coherent temporal framework. We hypothesise that if imaging data across scales and modalities can be systematically anchored to biologically meaningful temporal milestones, then disease trajectories can be reconstructed in a clinically actionable manner.

## Multiscale and multimodal advances still miss time

Over the past decade, biomedical imaging has made remarkable progress in both scale and depth. Techniques like high-resolution MRI, CT, and PET have improved clinical diagnosis by providing detailed views of internal structures and functional processes. In research, advances such as light-sheet fluorescence microscopy (LSFM), synchrotron-based hierarchical phase-contrast tomography (HiP-CT), and tissue clearing methods have enabled the three-dimensional (3D) imaging of intact organs at near-cellular resolution. There is a trend of combining these tools with molecular approaches including spatial transcriptomics, multiplexed immunofluorescence, and mass spectrometry imaging. Together, multiscale and multimodal methods (Table 1) offer a rich, hierarchical view of biology from gross anatomy to cellular phenotype and gene expression.

**Table 1:** Overview of multimodal and multiscale imaging technologies.

Category	Technology	Description	Advantages	Limitations	References
Multiscale strategies	HiP-CT	Stepwise zooming from organ to cellular resolution using synchrotron X-rays	Continuous 3D imaging from whole organ to micrometre scale; non-destructive	Requires synchrotron facility; limited accessibility; expensive	^4^
	micro-CT	Lab-based high-resolution X-ray imaging	Approaches histological detail; suitable for hard tissue	Less effective for soft tissue imaging	^5^
	Expansion Microscopy	Physical expansion of tissue for improved resolution	Achieves nanometre-scale imaging with standard microscopes	Applicable only to small samples or thin sections; limited field of view	^6^
	CLARITY	Hydrogel-based lipid removal method	Preserves protein/RNA; compatible with immunostaining	Slow; requires special setup; limited for large tissues	^7^
	iDISCO	Solvent-based rapid clearing approach	Fast; good for large samples; deep antibody penetration	Tissue shrinkage; less RNA-friendly; fluorescence loss	^8^
	CUBIC	Aminoalcohol-based clearing technique	High transparency; retains fluorescence; good for large tissues	Slower for dense samples; potential deformation	^9^
Multimodal strategies	PET/MRI	Simultaneously captures metabolic activity and anatomical structures	Combines high sensitivity (PET) with high resolution (MRI); enables functional and structural correlation	High cost; long acquisition time; technical complexity	^12^
	CODEX	Detects dozens of proteins within a single tissue section	High-resolution, multiplexed cellular data	Expensive; sensitive to staining quality	^14^
	Mass Spectrometry Imaging	Detects spatial distribution of metabolites or lipids	Label-free; useful for metabolic and drug distribution studies	Lower spatial resolution; complex interpretation	^15^
	Spatial Transcriptomics	Maps gene expression within tissue spatial context	Reveals cell heterogeneity and spatial relationships	High cost; limited resolution; complex data analysis	^16,17^

On the one hand, multiscale imaging technologies allow the same biological sample to be visualised across spatial hierarchies from organ level down to individual cells. Synchrotron-based HiP-CT can scan whole human organs (e.g. lung, brain, kidney) while zooming into micrometre-level subregions to capture local structural failure in disease progression^4^. Recent advances in laboratory-based phase-contrast micro-CT enable similar resolutions without requiring synchrotron access^5^. Optical expansion techniques such as expansion microscopy further extend resolution into the nanoscale domain^6^. Tissue clearing methods, including CLARITY^7^, iDISCO^8^, and CUBIC^9^, do not generate images themselves but make large, opaque samples transparent and antibody-permeable. When combined with LSFM, these approaches enable volumetric imaging of intact organs at cellular resolution^10,11^. For instance, Susaki et al. demonstrated whole-brain imaging in mice with single-cell detail^10^.

On the other hand, multimodal imaging integrates distinct biological data types within the same spatial framework. PET/MRI combine metabolic and anatomical information, improving tumour localisation and monitoring^12^. Multimodal workflows in research settings go further. Zhao et al.^13^ used tissue clearing and immunolabelling to probe whole human organs via LSFM, while also aligning results with clinical imaging. CODEX technology enables simultaneous visualisation of dozens of protein markers^14^. Mass spectrometry imaging maps the spatial distribution of metabolites^15^. Spatial transcriptomics adds gene expression landscapes on top of histological context^16,17^. Together, these methods bridge structural, molecular, and functional perspectives across biological systems.

Despite these achievements, most imaging studies still rely on cross-sectional analysis. They show where cells or molecules are located but not how they change over time. Even when timepoints are included, they are often few and poorly aligned. Longitudinal scans may vary in resolution, orientation, or imaging modality, making direct comparison difficult. Histological sections from different timepoints often involve different samples, introducing biological noise that limits conclusions.

This ‘snapshot mindset’ creates a fragmented understanding of disease. Researchers may observe key features but miss their order of appearance. Signals that appear important in late-stage samples might be consequences rather than causes. Without a temporal anchor, the dynamics of disease are flattened into static observations. Moreover, current imaging databases are usually structured by anatomical region or modality, not by sequence or trajectory. This makes it harder to reconstruct how a process unfolds or to model change in a meaningful way.

The gap is caused by practical and conceptual challenges. Imaging living systems over time is complex. It requires stable conditions, repeated access to the same sample, and high-resolution methods that do not cause damage. Even in animal models, these constraints limit the number and frequency of timepoints. In humans, ethical and technical barriers further restrict dynamic imaging, especially at microscopic scales. And while omics technologies have begun to map cellular states in developmental or disease trajectories, they often lack true temporal resolution. Pseudotime models infer change from static populations but cannot capture actual progression within a living system.

Despite these hurdles, the need to go beyond static snapshots is growing. We now have the tools to image structure, molecules, and function at high resolution. What we lack is a way to connect these images across time. Without that link, our understanding of disease remains partial, and it hinders the clinical translation of above-mentioned techniques.

## Technical and conceptual challenges in capturing temporal dynamics

Moving from spatial snapshots to temporal maps is not just a technical improvement. It requires rethinking how we capture, align, and interpret imaging data. At present, three core challenges stand in the way: fragmented data, missing time anchors, and disconnected platforms.

First of all, biomedical imaging data are still highly fragmented. Most imaging is designed for single-use interpretation. In clinical practice, scans are often performed using different machines, acquisition parameters, and protocols depending on the clinical purpose, such as diagnosis or follow-up. These variations make it challenging to perform consistent longitudinal comparisons. In research, even longitudinal datasets often involve different samples at each timepoint. This is especially true in human tissue studies, where repeated sampling is limited. As a result, we are often left comparing separate images that do not form a continuous timeline.

Most imaging lacks proper temporal anchoring. Without consistent reference points, even high-quality data cannot be meaningfully aligned across time. In developmental biology, time is often inferred from morphological stage. In disease studies, it may be based on symptom onset or treatment schedule. But these markers are coarse and variable. Imaging needs more precise anchors. Specifically, biological or technical cues that allow images to be matched across stages. These could include anatomical landmarks, spatial gene expression patterns, or physiological signals.

Imaging platforms remain largely isolated. Each modality (MRI, PET, CT, LSFM, HiP-CT) has its own hardware, software, and data format. While multimodal imaging has made progress in integrating signals at one timepoint, integrating across time remains rare. The lack of standardised workflows and interoperable file formats makes it hard to combine scans from different systems. Even within the same modality, changes in resolution, orientation, or noise levels can disrupt alignment. In multiscale imaging, there is no unified framework for linking data in time.

These challenges can be tackled. The field has already made progress in spatial alignment, cross-platform registration, and metadata standards. Extension into the temporal domain is what we need for the next stage. Temporal sequences must be designed in imaging protocols, not just space. Metadata must include timing information that allows images to be matched and compared. And computational methods must be developed to handle change across timepoints. Without addressing these issues, dynamic disease mapping will remain out of reach.

## Principals for temporal atlas of disease

Unlike isolated snapshot data, a temporal atlas systematically integrates information across spatial scales, biological modalities, and clinically relevant timepoints. We propose that future efforts focus on three essential principals: data stacking, temporal anchoring, and change modelling. Each needs to be applied across time, modality, and scale.

Time as a fourth dimension faces several challenges. Longitudinal scans are often inconsistent, most datasets lack reliable temporal anchors, and multimodal or multiscale data are difficult to align across time. In practice, uniformly high spatiotemporal imaging is not always necessary. Low-resolution, high-frequency imaging can be used for continuous monitoring, and to acquire high-resolution imaging only at key transition points.

Stacking multiscale and multimodal data across time can reconstruct the true trajectory of pathology. Rather than analysing each modality or resolution in isolation, researchers should build longitudinal stacks that combine repeated measurements across different biological modalities. For example, a liver fibrosis atlas might include serial MRI scans showing macroscopic architectural changes; PET data tracing fibrotic activity; spatial transcriptomics of ECM gene expression in biopsy cores; and LSFM imaging of immune cell dynamics. These data must be acquired at multiple timepoints and linked across anatomical landmarks to track disease evolution.

Anchoring data through shared biological references is essential to register different datasets across time, modality, and scale. It allows longitudinal and multimodal comparisons. Temporal anchors define when a sample or image fits in the progression of disease (e.g., symptom onset, treatment response, or molecular thresholds like collagen density). Modality anchors help align images from different platforms by using shared spatial features (e.g., vasculature, organ boundaries). Scale anchors allow data collected at different resolutions (e.g., MRI vs. histology) to be spatially integrated.

Modelling dynamic biological change transforms static data into predictive insight. Once data are stacked and anchored, temporal atlases must be computationally modelled to reconstruct intermediate states, predict future changes, and infer causality. This requires algorithms capable of translating across scales and modalities. Recent advances in deep-learning frameworks now enable high-throughput mass spectrometry to reconstruct fine biochemical patterns across entire brain sections^18^. By linking molecular signals at the cellular level with tissue-scale architecture, these methods offer a multiscale view of spatial organisation. However, such integration remains focused on static snapshots. To reveal how diseases evolve, similar algorithms must be extended to align changes over time.

Non-destructive imaging can also be complemented by virtual histology, where deep learning models enable longitudinal inference of histological events across time^19^. In addition, transferring models across species may help align temporal patterns between basic research and clinical imaging, supporting reverse translation and improving the continuity of temporal assessments^20^. These approaches expand the information that can be incorporated into a temporal atlas without repeated destructive procedures.

Constructing such atlases requires coordinated efforts across disciplines. Imaging protocols should emphasise longitudinal consistency and robust metadata collection. Data repositories need to be designed with temporal relationships in mind, while bioinformatics pipelines ought to facilitate alignment, registration, and meaningful interpretation. Open access also plays a critical role in enhancing transparency, reproducibility, and the translation of research into clinical practice.

## From vision to implementation

Rather than proposing a specific algorithm or a disease-specific temporal model, the following sections outline a practical roadmap for implementing temporal atlases across imaging modalities, spatial scales, and biological contexts. The construction of a temporal atlas is not only a scientific ambition but a practical necessity. To achieve it, the imaging community must align efforts across three key aspects: data acquisition, standardisation, and collaborative infrastructure.

1. Standardised longitudinal imaging protocols: while existing studies may span early, middle, and late stages of disease, these timepoints are often selected opportunistically, driven by clinical availability rather than biological logic. Imaging is typically performed at diagnosis, during treatment, or at follow-up, but seldom aligned with the underlying dynamics of pathology, such as the onset of cellular senescence, immune infiltration, or fibrotic remodelling. In both clinical and research contexts, this demands harmonised imaging windows, standardised acquisition settings, and explicit annotation of disease stages from the outset. Such a shift moves biomedical imaging from a collection of static snapshots toward a dynamic framework capable of capturing progression. It also lays the foundation for downstream applications such as trajectory modelling, personalised prognosis, and time-aware AI models. Temporal sampling does not need to be uniform. Chronic diseases may only require sparse imaging on key clinical milestones, whereas rapidly progressing conditions may need more frequent imaging around suspected transition phases. Multicentre and longitudinal imaging also require consistent standards across machines and sites. Practical strategies include harmonised acquisition parameters, routine phantom-based quality control, and simple computational normalisation methods such as intensity standardisation or histogram matching. These procedures are widely used in many multicentre studies without requiring identical hardware. When technology evolves, consistency can be preserved by scanning shared phantoms before and after upgrades. Recording reconstruction and metadata parameters also improves harmonisation.
2. Establishment of time-aware standard and anchors: to support temporal integration, imaging datasets must change from isolated snapshots to structured trajectories. A key step is the definition of biologically meaningful time anchors using reference points such as symptom onset, peak inflammation, therapeutic response, or early molecular shifts. Current studies often rely on opportunistic sampling, aligned with clinical schedules or model availability rather than pathological logic. This leads to fragmented timelines and inconsistent interpretations. True temporal anchoring does not require absolute synchronisation across patients or animal models. Instead, it enables semantic alignment, where datasets collected at different times or conditions can still be compared based on shared biological milestones. In animal studies, induction protocols allow for planned sampling, and retrospective analysis can assign anchors post hoc using molecular or structural indicators. In clinical settings, anchors may be defined by standard events (e.g., diagnosis, treatment initiation), complemented by clinical scores, imaging features, or molecular biomarkers. Standardising these anchors and embedding them in metadata lays the foundation for longitudinal comparison across platforms and studies. Initiatives like the Human Cell Atlas and Allen Brain Atlas have introduced shared schemas, but few include time. Establishing such standards will enable multiscale and multimodal datasets to be synchronised in both space and time, supporting more coherent analyses.
3. Development of tools and platforms for temporal modelling: analysis tools and public databases are expected to support longitudinal alignment, not just cross-sectional querying. Platforms should enable the stacking of heterogeneous data, registration across modalities, and interpolation across timepoints. Open-source libraries, federated data models, and user-friendly visualisation tools will be essential. These platforms may support both biological discovery and clinical translation, allowing clinicians to compare a patient’s data trajectory against disease atlases derived from population-level dynamics.
4. Encouragement of cross-disciplinary collaboration: dynamic imaging is at the intersection of radiology, microscopy, computational modelling, systems biology, and clinical practice. Funding bodies should prioritise projects that bridge these domains. Training programmes should prepare researchers to work across experimental and computational environments. Ethics frameworks must adapt to protect patient identity in longitudinal, high-dimensional datasets, while also supporting data sharing and re-use.

Realising a temporal atlas of disease requires more than technological innovation but calls for shared standards, open collaboration, and a sustained commitment to turning time into a dimension of human health.

## Data Availability

No datasets were generated or analysed during the current study.
